# Supplementary material for: Real-time monitoring of hydrophobic aggregation reveals a critical role of cooperativity in hydrophobic effect
Source: Nat Commun. 2017 May 31;8:15639. doi: 10.1038/ncomms15639 (PMC5460034; doi:10.1038/ncomms15639)
Supplement: Supplementary Information — Supplementary Figures, Supplementary Tables, Supplementary Notes and Supplementary References [file ncomms15639-s1.pdf]

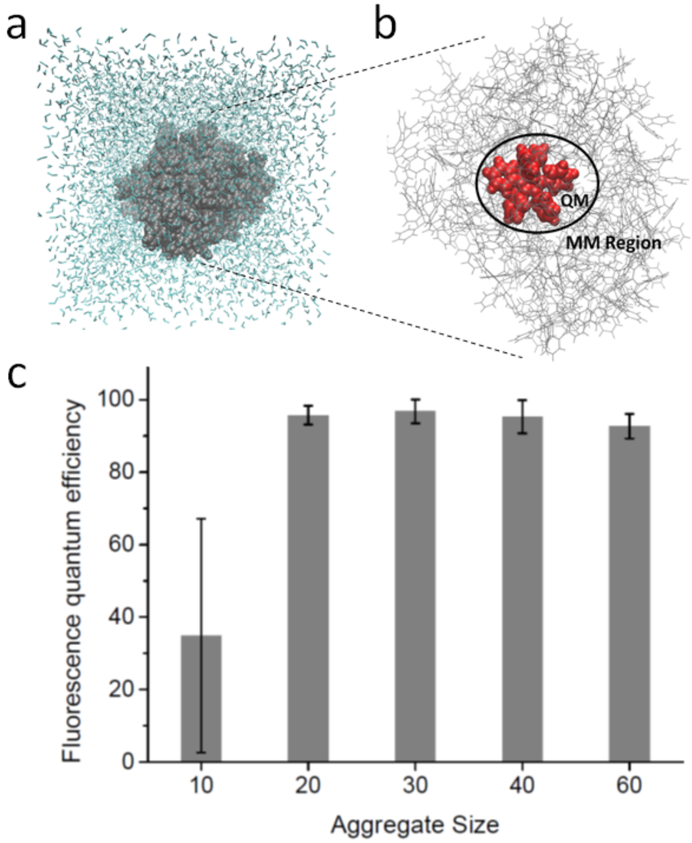

Supplementary Figure 1 | Fluorescence quantum efficiency of HPS amorphous aggregates. (**a**) A representative amorphous aggregate containing 60 HPS molecules obtained from MD simulations. HPS molecules and water molecules are shown in gray and cyan color, respectively. (**b**) The quantum-mechanics/molecular-dynamics (QM/MM) model: the centered HPS molecule in red color is treated as the QM region, and the surrounding 59 HPS molecules are treated as the MM region as the environment for the QM region. (**c**) The fluorescence quantum efficiency (FQE) of HPS molecule in aggregate containing different number of molecules calculated from QM/MM simulations. This figure is plotted based on the data reported in Ref [1].

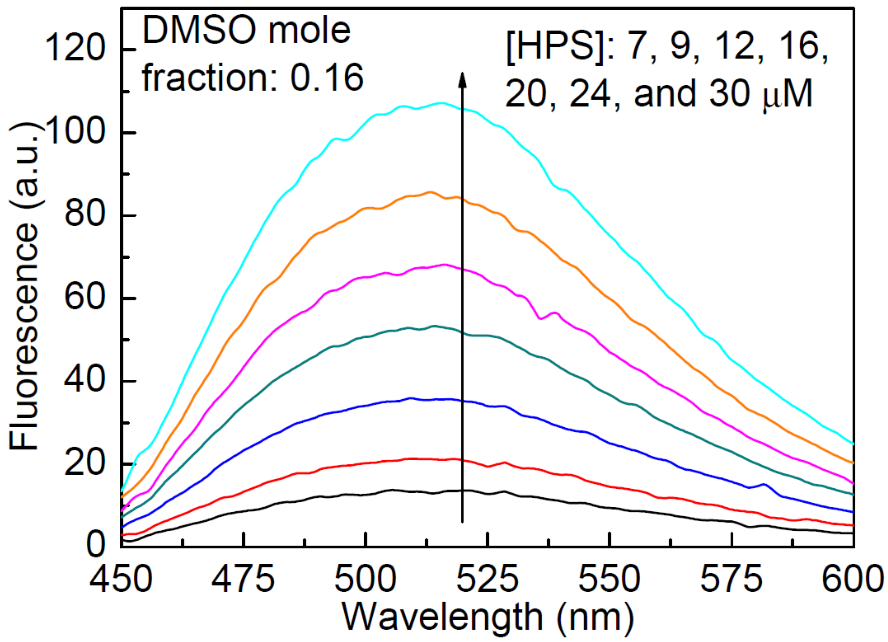

Supplementary Figure 2 | Emission spectra of HPS aggregates. This figure shows the emission spectra of HPS aggregates in solution at various HPS concentrations (λ_{ex}= 405 nm). The solvent is the DMSO/water mixture with a DMSO mole fraction of 0.16.

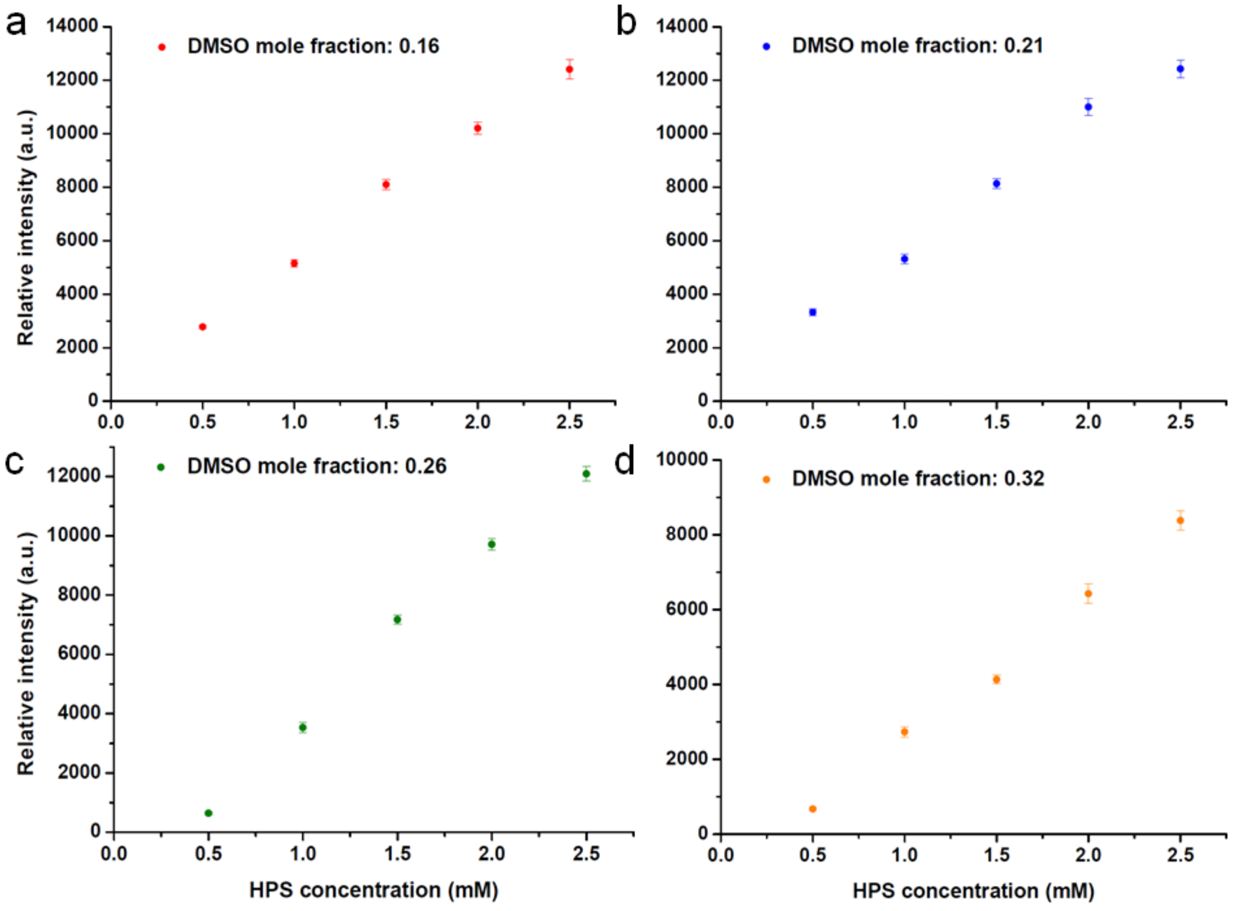

Supplementary Figure 3 | Fluorescence intensity vs. total volume of aggregates in bulk solvents. This figure shows the fluorescence of HPS aggregates at various HPS concentrations and in various DMSO/water solvent mixtures measured by spectrofluorometer. The solvent mixtures have a DMSO mole fraction of (**a**) 0.16, (**b**) 0.21, (**c**) 0.26, and (**d**) 0.32. (λ_{ex}= 405 nm, λ_{em}= 510 nm)

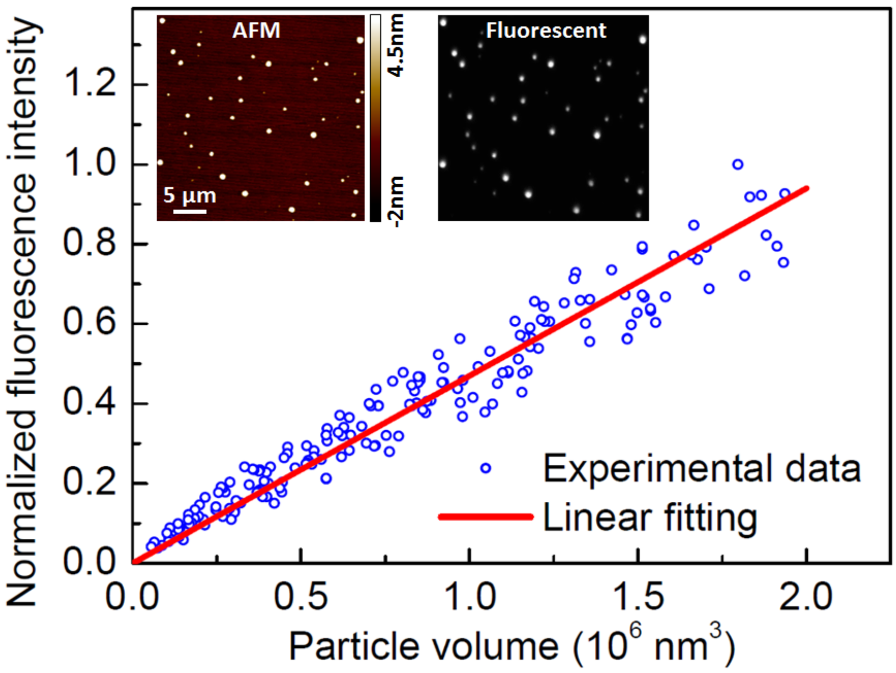

Supplementary Figure 4 | Fluorescence intensity is linear to the volume of HPS aggregates. The volume (inset, AFM image) and fluorescence (inset, fluorescence image) of individual HPS aggregate measured by fluorescence confocal atomic force microscopy. Red solid line represents the linear fitting of experimental data.

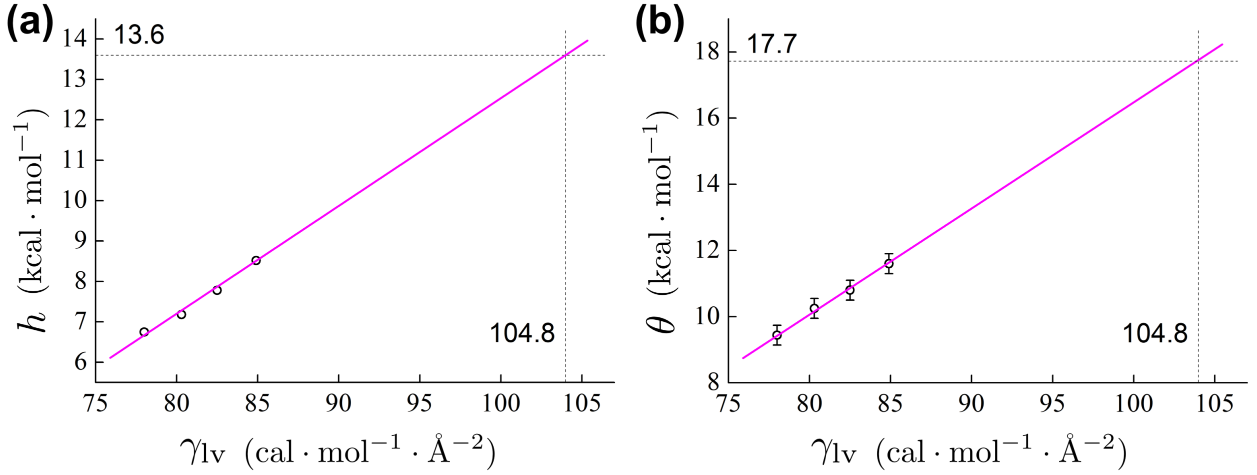

Supplementary Figure 5 | Linear extrapolation of h and γ_{sl} for HPS aggregation in pure water. (**a**) Linear relationship between h and the surface tension of corresponding solvent γ_{lv}. (**b**) Linear relationship between the solute-solvent surface tension of HPS aggregates θ and the surface tension of corresponding solvent γ_{lv}. The error bars are represents the error of fitting θ which is given by Supplementary Figure 11.

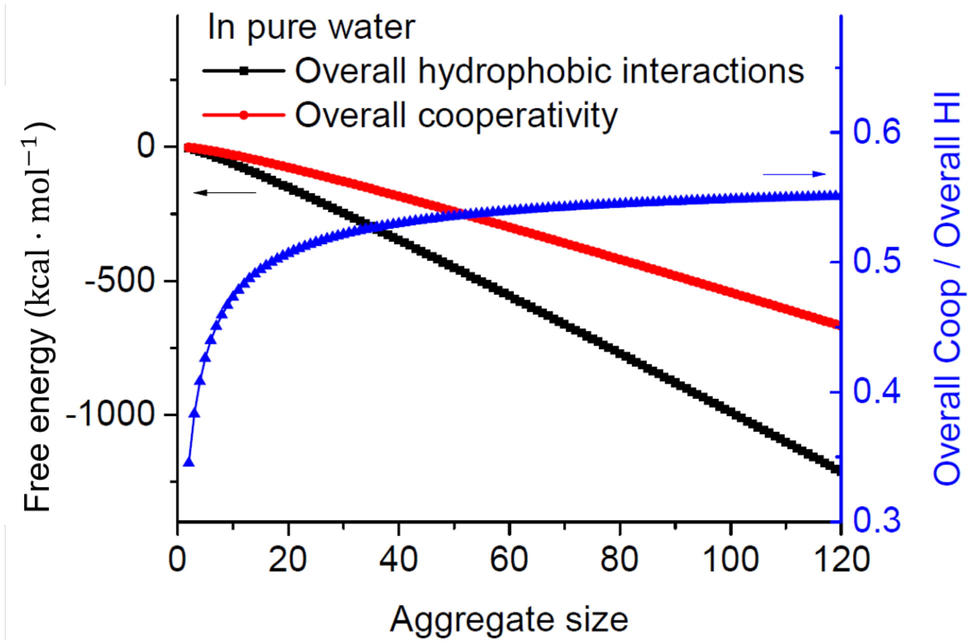

Supplementary Figure 6 | Free energy and cooperativity of HPS aggregation in pure water. Red line with square symbols shows the accumulated cooperativity data in Figure 1c; black line with dot symbols shows the accumulated $\Delta\Delta G$ data in Figure 1c; and blue line with triangle symbols shows the ratio between the accumulated cooperativity and the accumulated $\Delta\Delta G$.

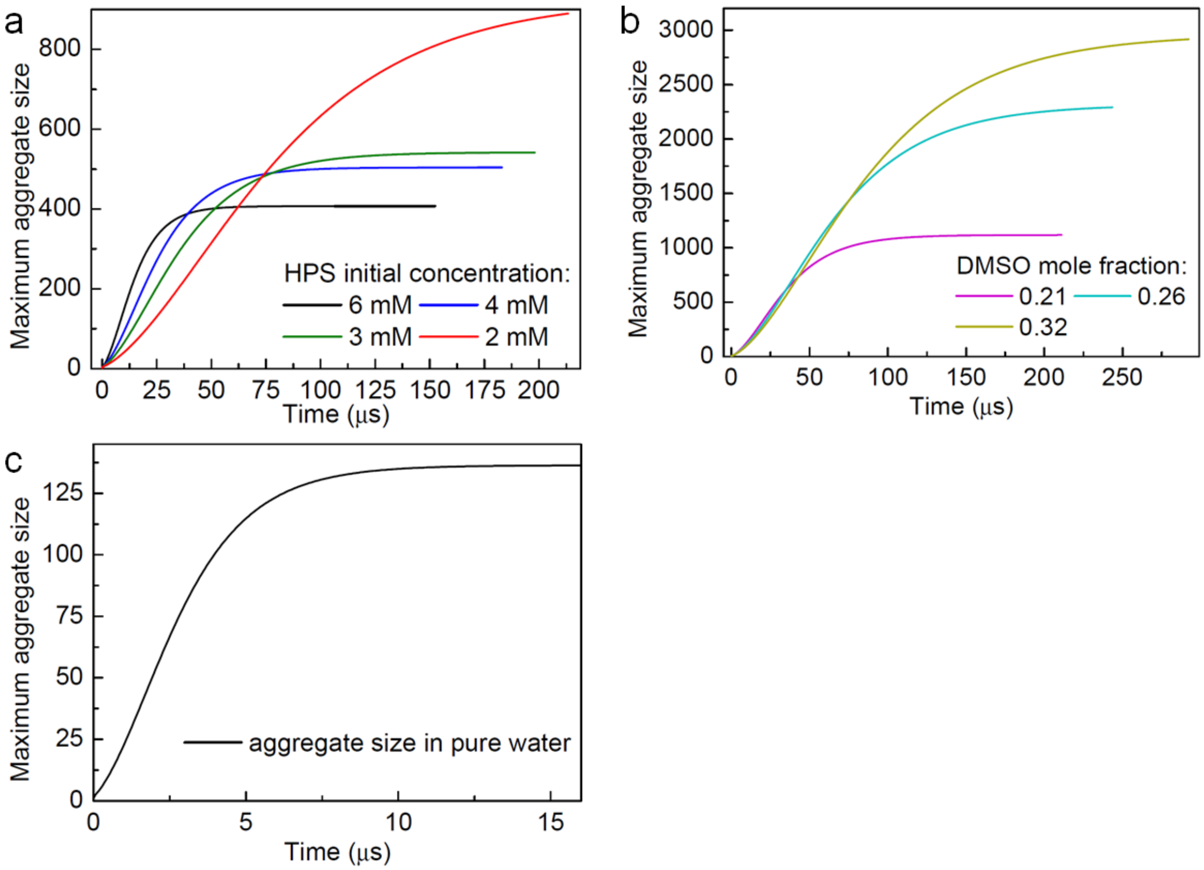

Supplementary Figure 7 | Maximum aggregate size as a function of time. (**a**) Various HPS initial concentrations in the DMSO/water mixture with a DMSO mole fraction of 0.16. (**b**) Various DMSO/water solvent conditions with a HPS initial concentration of 6 mM. **c,** Pure water condition with a HPS initial concentration of 6 mM.

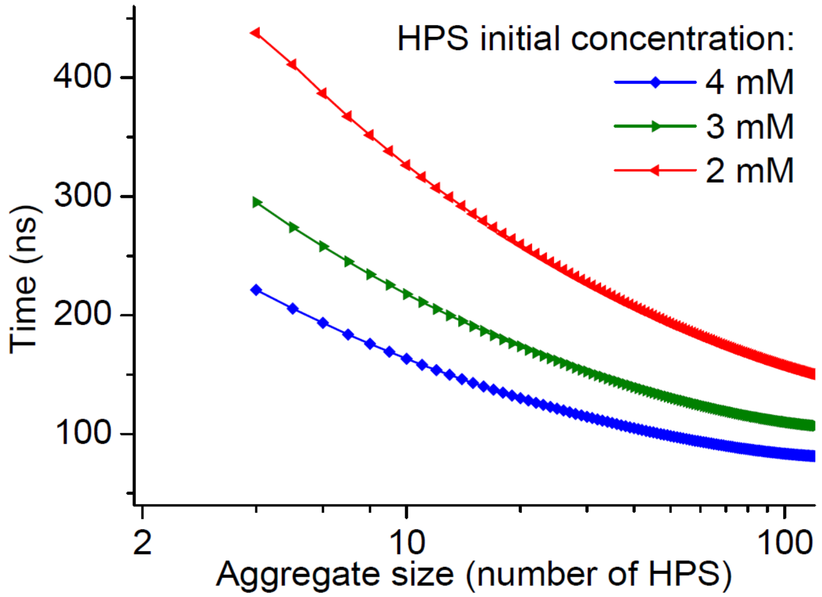

Supplementary Figure 8 | The monomer attachment time to a HPS aggregate. The monomer attachment time presented here is calculated for various HPS initial concentrations. The solvent is the DMSO/water mixture with a DMSO mole fraction of 0.16.

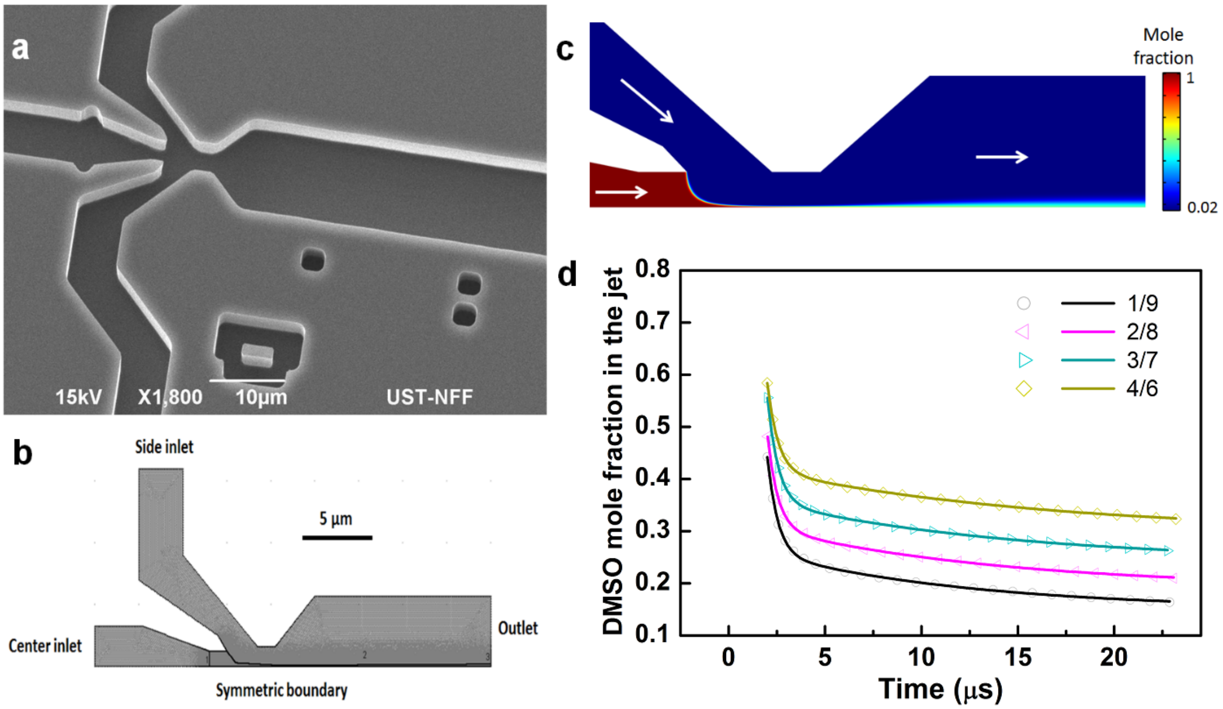

Supplementary Figure 9 | The design, fabrication and characterization of the microfluidic mixer. (**a**) A SEM image of the fabricated microfluidic mixer on a silicon substrate. (**b**) The symmetric 2D model of the mixer with meshing elements used in computational fluid dynamics simulations. The model has two inlets, one outlet, a symmetric boundary, and surface walls for other boundaries. (**c**) A color map shows a representative simulation result with DMSO/water volume ratio of 1/9 in the side stream. (**d**) Simulation results showing the rapid depletions of DMSO in the hydrodynamically sheathed stream at all conditions (in symbols). Labels for different colored symbols represent different DMSO/water volume ratios in the side stream. The solid lines correspond to results of fitting the DMSO mole fraction change as a function of time to double-exponential functions at various experimental conditions.

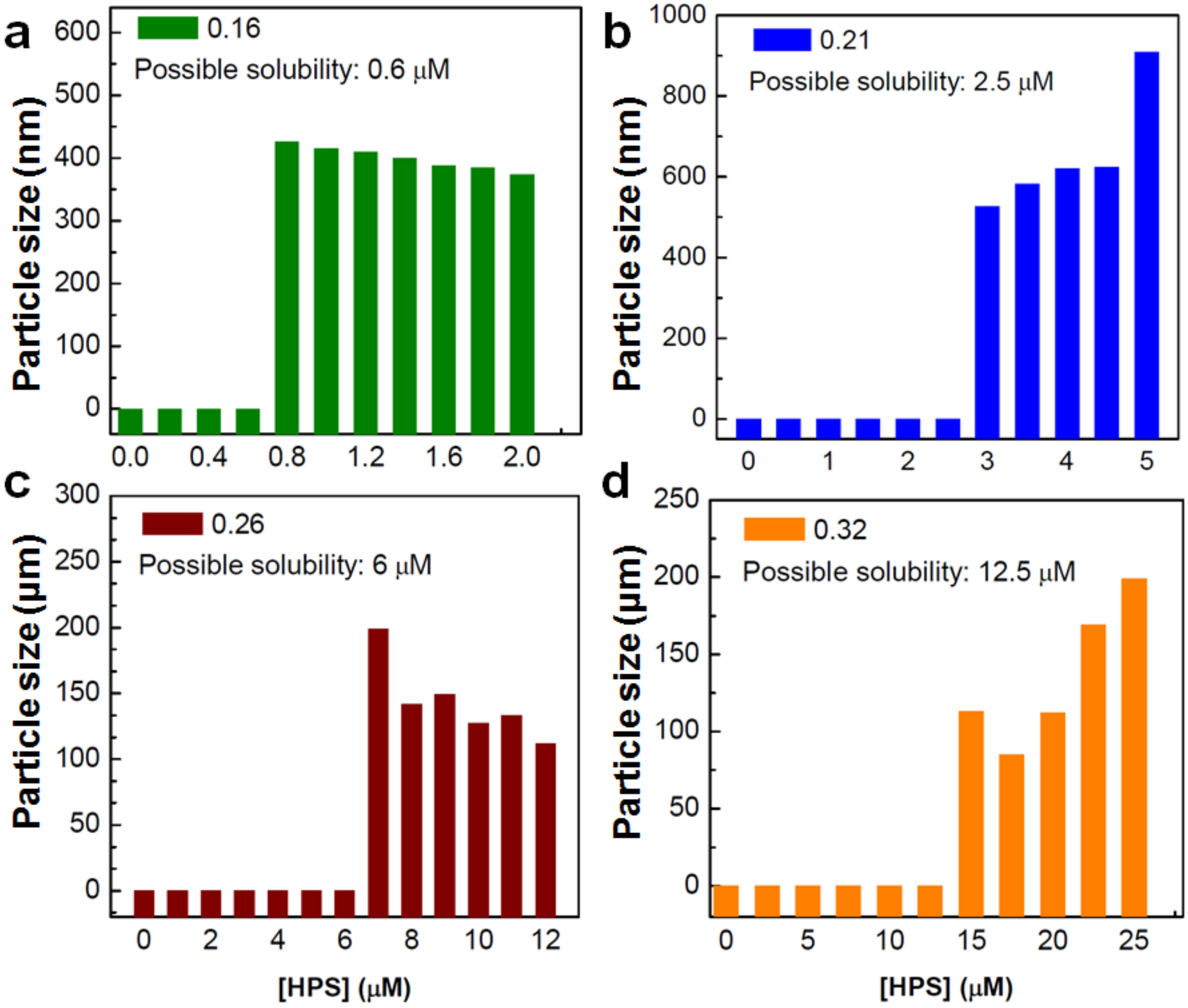

Supplementary Figure 10 | Solubility measurement of HPS in water/DMSO mixtures. The solubility presented here are measured for the DMSO mole fractions of (**a**) 0.16, (**b**) 0.21, (**c**) 0.26, and (**d**) 0.32. Each colored bar in a figure represents the result of particle size analysis of the bulk solution with a specific HPS concentration. The corresponding possible solubility is indicated.

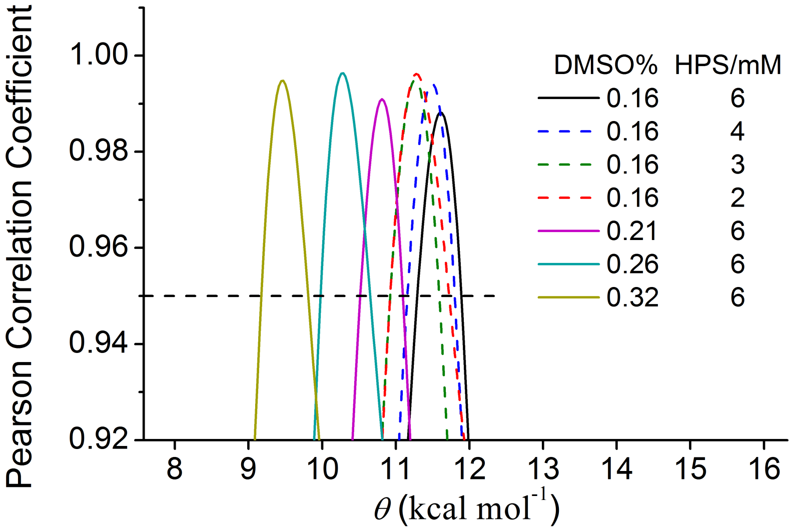

Supplementary Figure 11 | Estimation of the fitting error of θ. With the Pearson correlation coefficients for each theoretical fittings, the standard deviation of the fitting error of θ is ± 0.3 kcal $\cdot$ mol^{-1} with the Pearson correlation coefficient (r) threshold of 0.95. The dashed horizontal line shows the threshold of $r = 0.95$.

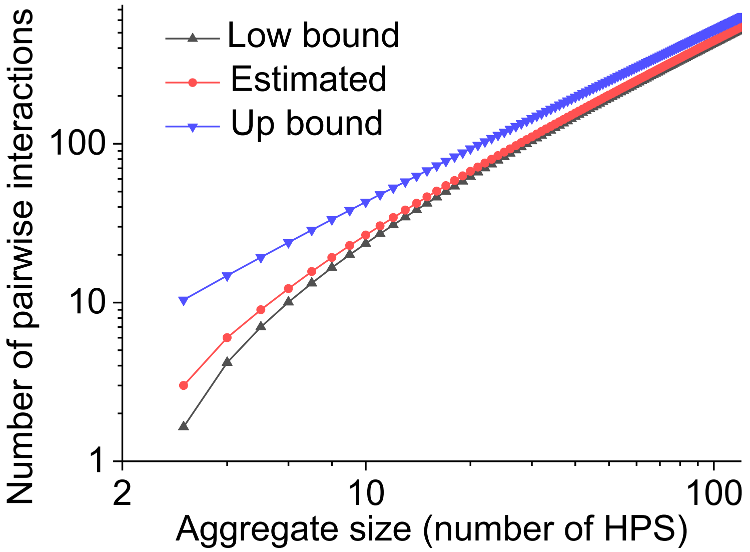

Supplementary Figure 12 | Adopted values of contact pair number and their bounds. The estimated pair numbers are calculated from equation (12).

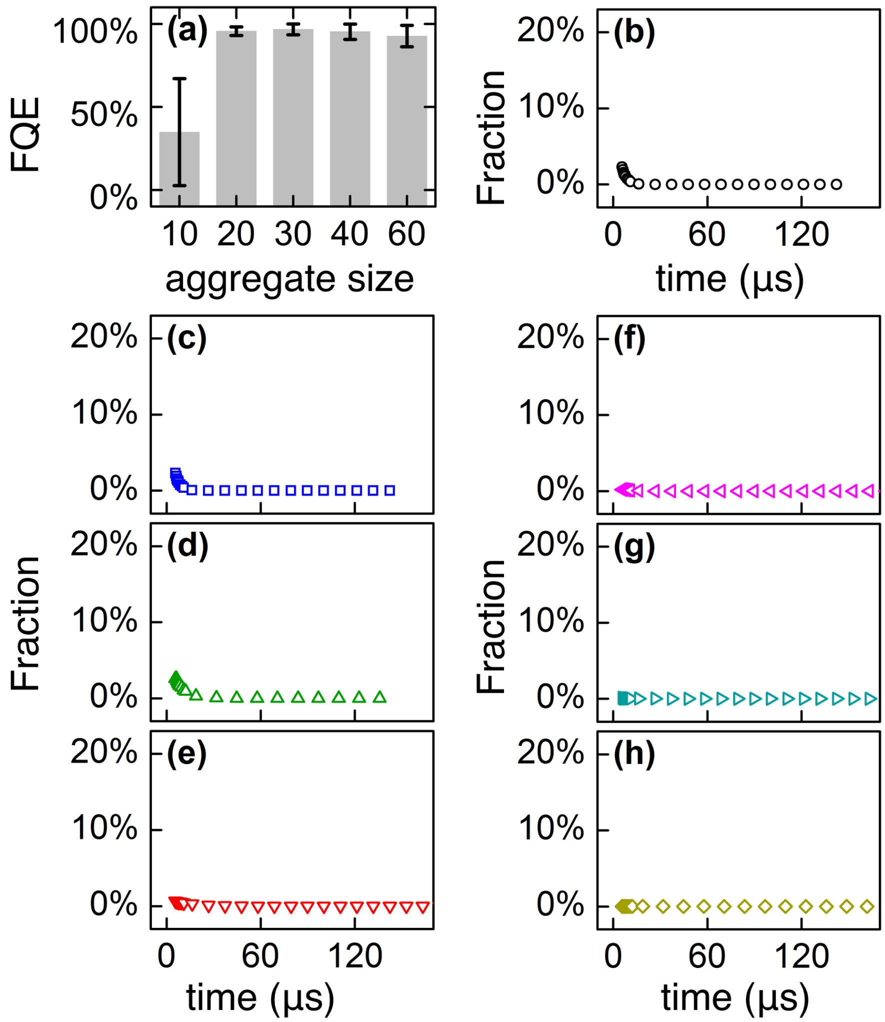

Supplementary Figure 13 | Volume fractions of small aggregates (<20) to all aggregates. (**a**). The fluorescence quantum efficiencies (FQEs) of HPS amorphous aggregates with sizes of 10, 20, 30, 40 and 60 were compared, where the error bars are the standard deviation of the FQEs of 5 different conformations for each data point. This figure is plotted based on the data reported in Ref [1]. (**b-h**). The fraction of the small aggregates ($n < 20$) in total aggregate volume as a function of time for seven experimental systems in our study. The black, blue, green and red curves in (**b-e**) represent the systems in the same solvent (with 0.16 mole fraction of DMSO) but with different initial HPS concentrations of 6 mM, 4 mM, 3 mM and 2 mM, respectively; while the black, magenta, cyan and yellow curves in (**b**), (**f-h**) represent the systems with the same HPS initial concentration (6 *mM*), but with different DMSO mole fractions of 0.16, 0.21, 0.26 and 0.32, respectively.

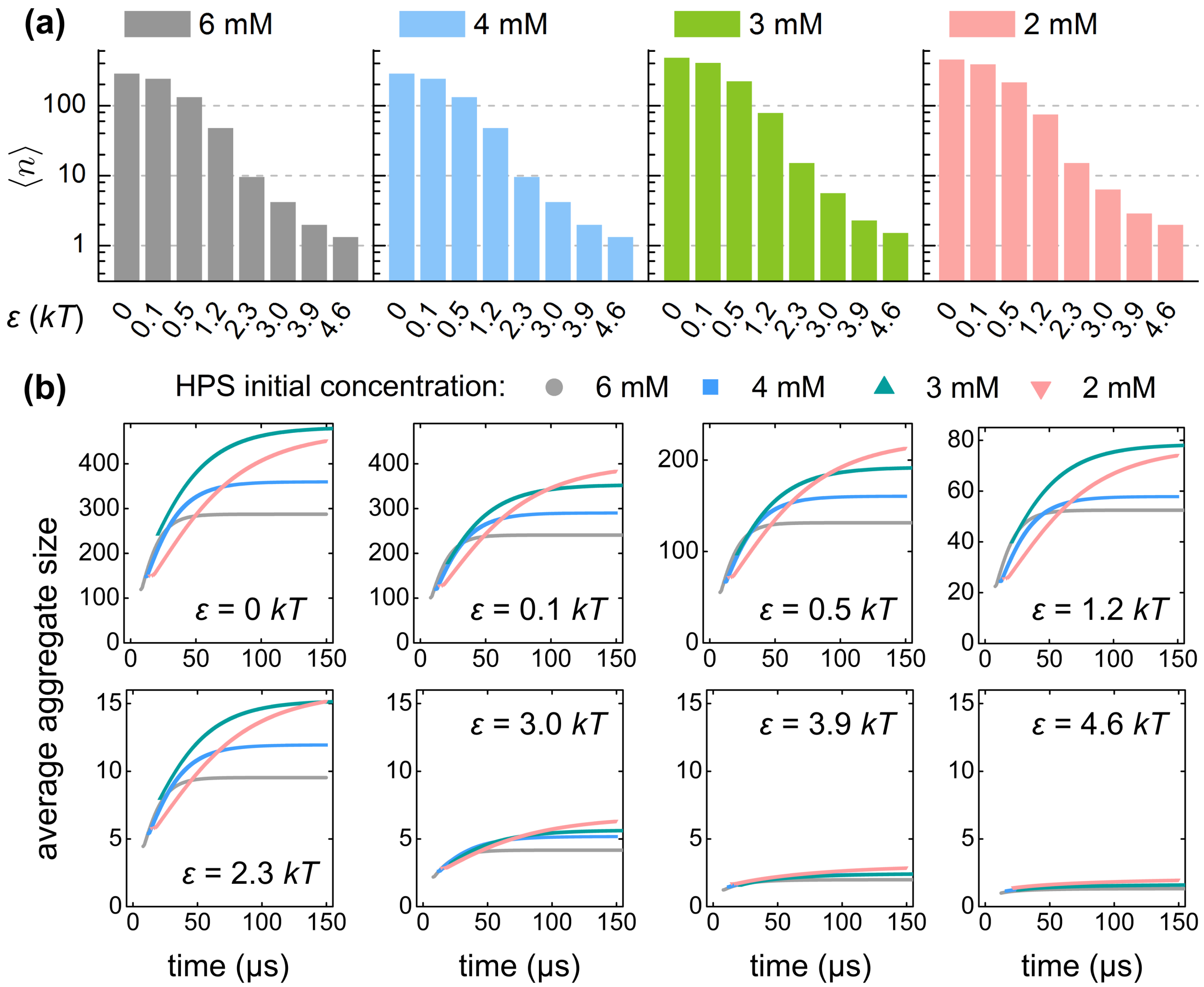

Supplementary Figure 14 | Aggregate sizes predicted from non-CNT at different values of ε. (**a**). The averaged aggregate size $\langle n \rangle$ at the end of the microfluidic tube ($t = 150\ \mu s$) obtained from the non-CNT model with various values of ε ($\varepsilon = 0, 0.1, 0.5, 1.2, 2.3, 3.0, 3.9,$ and $4.6\ kT$) under experimental conditions with different initial HPS concentrations ([HPS] $= 6, 4, 3,$ and 2 mM). When ε =0 kT, the non-CNT model is equivalent to a CNT model. (**b**). The same as (**a**) except that the average aggregate size as a function of time is displayed.

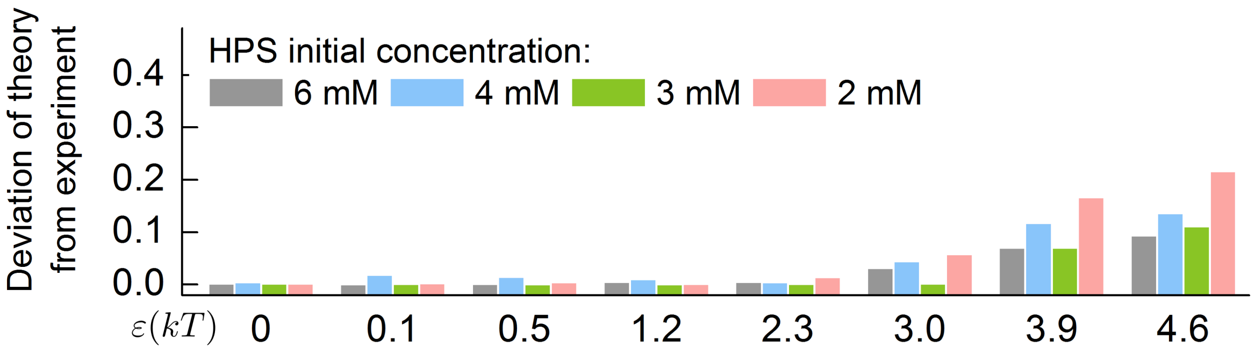

Supplementary Figure 15 | Fitting errors of non-CNT theories at different values of ε. The deviation of theoretical fitting from experiment on the slope of the fluorescence curve: $\delta = \langle\left(\dot{I}_{\text{exp}}(t) - \dot{I}_{\text{theory}}(t)\right)/\dot{I}_{\text{exp}}(t)\rangle$. The non-CNT model was applied for the theoretical fitting with various values of ε ($\varepsilon = 0, 0.1, 0.5, 1.2, 2.3, 3.0, 3.9,$ and $4.6\ kT$) under experimental conditions with different initial HPS concentrations ($[HPS] = 6, 4, 3,$ and 2 mM). Specifically, segments of fluorescence curves, $0.5 < I < 0.95$, which cover major parts of the aggregate growth were taken into account to calculate the deviation.

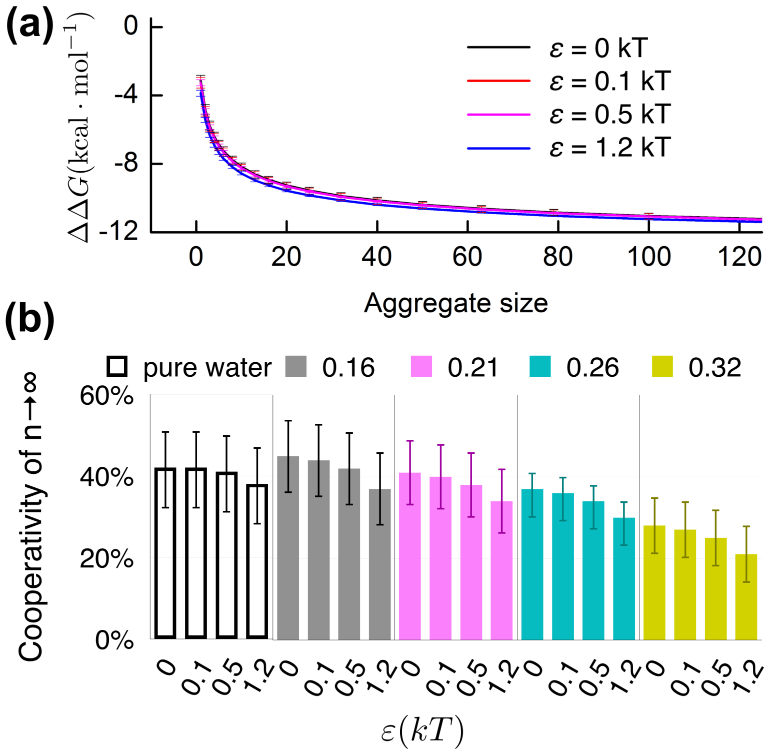

Supplementary Figure 16 | $\Delta\Delta G$ and cooperativity calculated from non-CNT theories. Hydrophobic free energy ($\Delta\Delta G$) and cooperativity computed from the non-CNT model with different values of ε. (**a**) $\Delta\Delta G$ of HPS aggregation in pure water when ε is 0 (black, reduced to the CNT model), 0.1 (red), 0.5 (magenta) and 1.2 (blue), respectively. The error bars show the range of $\Delta\Delta G$ that are resulted from the fitting error of θ. (**b**) Cooperativity of attaching a HPS molecule to an infinite sized HPS aggregate, where the black box, grey, magenta, green and yellow bars represent the systems in pure water, DMSO/water solvent mixtures with 16%, 21%, 26% and 32% of DMSO, respectively. The error bars show the range of cooperativity calculated from $\theta - \Delta\theta$ to $\theta + \Delta\theta$, where $\Delta\theta = 0.3\ \mathrm{kcal \cdot mol^{-1}}$ is the fitting error of θ.

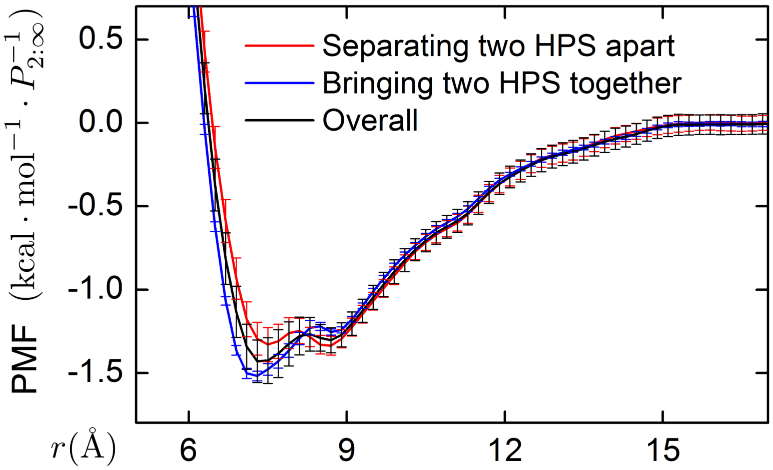

Supplementary Figure 17 | Potential of mean force of the aggregation of a HPS dimer in pure water. The potential of mean force (PMF) per molecular contact ($P_{2:\infty}$, the number molecular contact of HPS dimer with the same strength as the molecular contacts in the infinite size aggregate) of bringing two HPS molecules together in water solution is obtained from umbrella sampling molecular dynamics (MD) simulations. PMF curves from two independent sets of simulations are reported: one was bringing two HPS molecules together from 20 to 5 Å in Si-Si distance (in blue), while the other one was separating two molecules apart from 5 to 20 Å (in red). The window width of the umbrella sampling is chosen to be 1 Å, and the restraint force constant was set to be 4.78×10^4 kcal · mol^{-1}Å^{-2}. The error bars of the PMF are obtained from 10 bootstraps of the umbrella sampling data.

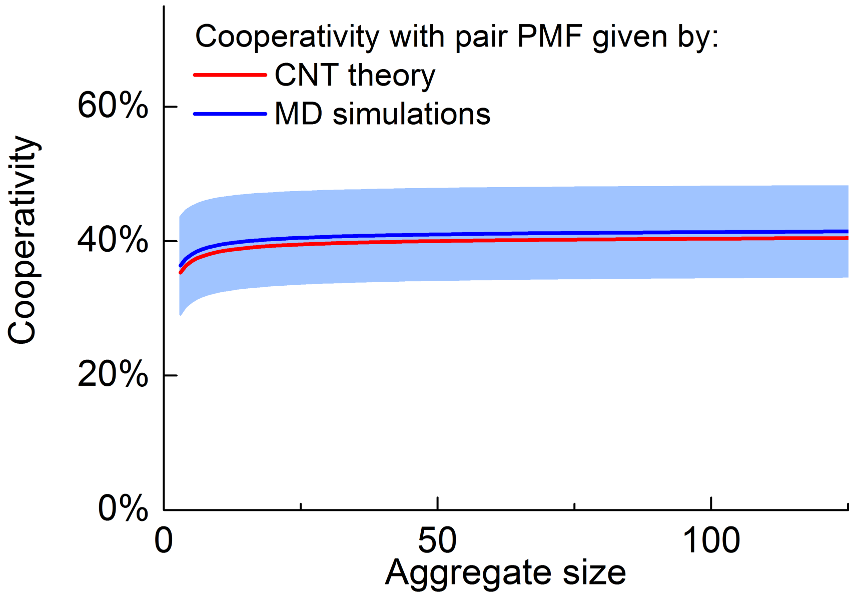

Supplementary Figure 18 | Cooperativity with pairwise interaction computed from MD. The contribution of cooperativity to hydrophobic free energy as a function of aggregate size are displayed. Results from the CNT theory with pair potential of mean force (δF) estimated by supplementary equation 14 are plotted in red, while results with δF and its uncertainty computed from umbrella sampling MD simulations are displayed in blue.

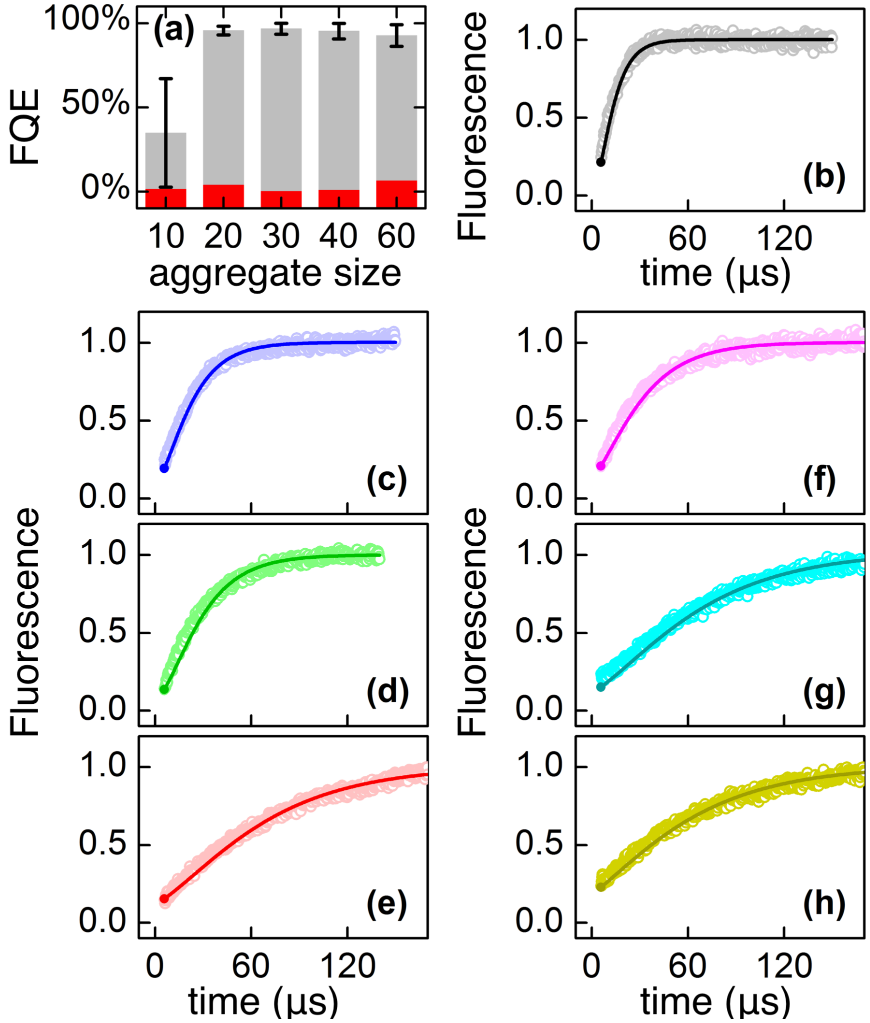

Supplementary Figure 19 | Theoretical fluorescence intensity considering only fully buried HPS. (**a**) shows the fluorescence quantum efficiencies (FQEs) of HPS amorphous aggregates with sizes of 10, 20, 30, 40 and 60 were compared. The gray and red bars represent for the QFE of buried and exposed HPS molecules, respectively. The error bars represent the standard deviation of the FQEs of 5 different conformations for each data point. This figure is plotted based on the data reported in Ref [1]. (**b-h**) are the total fluorescence intensity calculated from the theory that only considers the fluorescence of fully buried HPS molecules. Using the total volume of embedded HPS molecules to fit with the experimental data generates the same results as using the total volume of all HPS molecules in aggregates. The light dots are the experimental results and the solid curves represent the predictions of the theory. The black, blue, green and red curves in the upper row represent the systems with initial HPS concentrations of 6 mM, 4 mM, 3 mM and 2 mM respectively; while the black, magenta, cyan and yellow curves of the bottom row represent the systems with DMSO concentrations of 0.16, 0.21, 0.26 and 0.32, respectively. The error bars in (**a**) are the standard deviations of the FQEs of 5 different conformations for each data point.

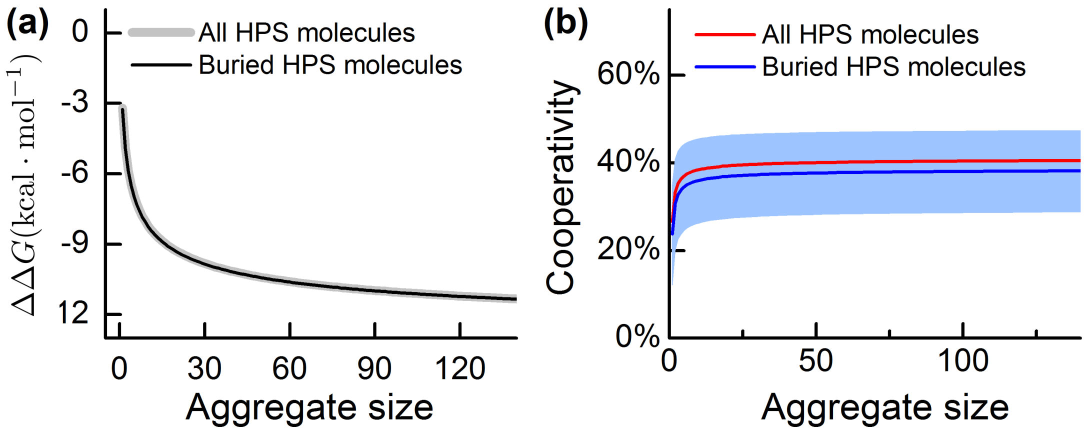

Supplementary Figure 20 | $\Delta\Delta G$ and cooperativity computed from the fully-buried model. $\Delta\Delta G$ and cooperativity are calculated by the fittings to the experimental fluorescence intensity with the theoretical volumes of all the aggregates (the thick gray curve in (**a**) and the red curve in (**b**) and the embedded HPS molecules (black curve in (**a**) and the blue curve in (**b**)).

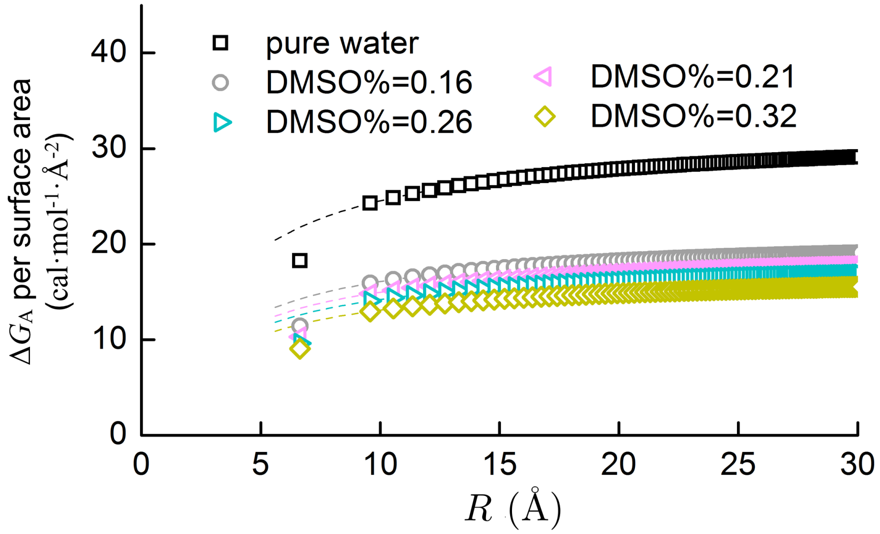

Supplementary Figure 21 | Solvation free energy (ΔG_A) as a function of the solute radius. The black, gray, magenta, blue and green dots represent the systems with DMSO mole fraction of 0, 0.16, 0.21, 0.26 and 0.32, respectively. The first point at $R = 5.9$ Å corresponds to the solvation free energy an individual HPS molecule (h) divided by its solvent accessible surface area (750 Å^2). The other points correspond to HPS aggregates with size three and above, and their hydration free energies and surface area were obtained from $\theta n^{2/3}$ and $4\pi(R_n + R_W)^2$, respectively. Please refer to Supplementary Note 7 for calculation details. The dashed lines are the extrapolation of all the points after the crossover of the length scale (aggregates of size 3 and above with $R > 10$ Å). These lines are all above the first point before the crossover ($R = 5.9$ Å), indicating a kink before and after the crossover of the length scale as predicted by the LCW theory.

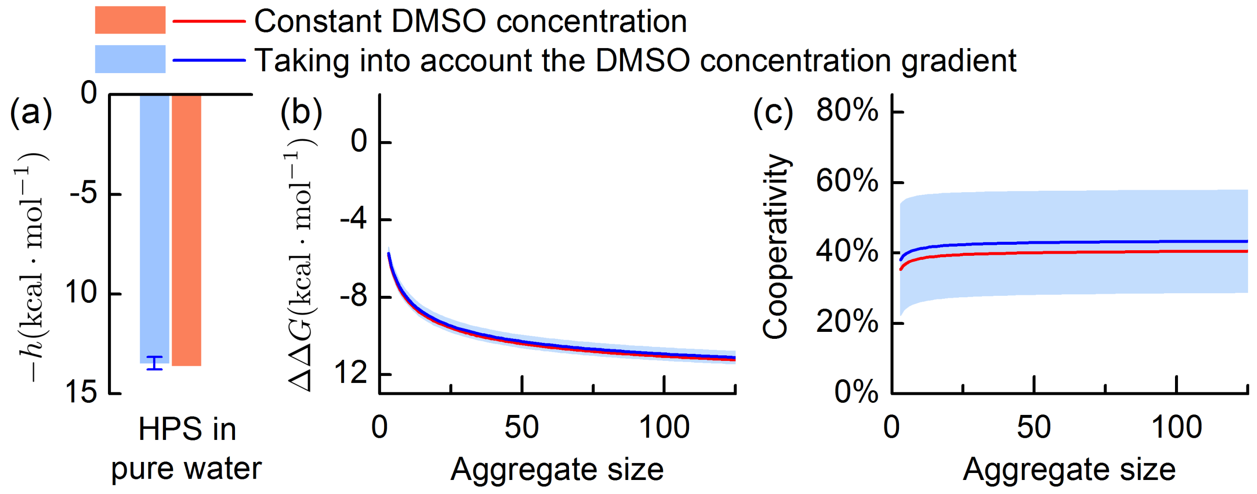

Supplementary Figure 22 | $\Delta\Delta G$ and cooperativity considering the DMSO concentration gradient. (**a**). Comparison of phase transfer free energy ($-h$) computed from the original model with constant DMSO concentration (in blue) and a modified model considering DMSO concentration changing along the microfluidic tube (in red). The uncertainty of the blue bar was estimated from the standard deviations of $-h$ values along the microfluidic tube. (**b**) and (**c**) show $\Delta\Delta G$ and cooperativity, respectively for the original model (blue curves and bars) and the modified model (red curves and bars). The uncertainties of blue curves were computed based on the uncertainty of $-h$ reported in (**a**).

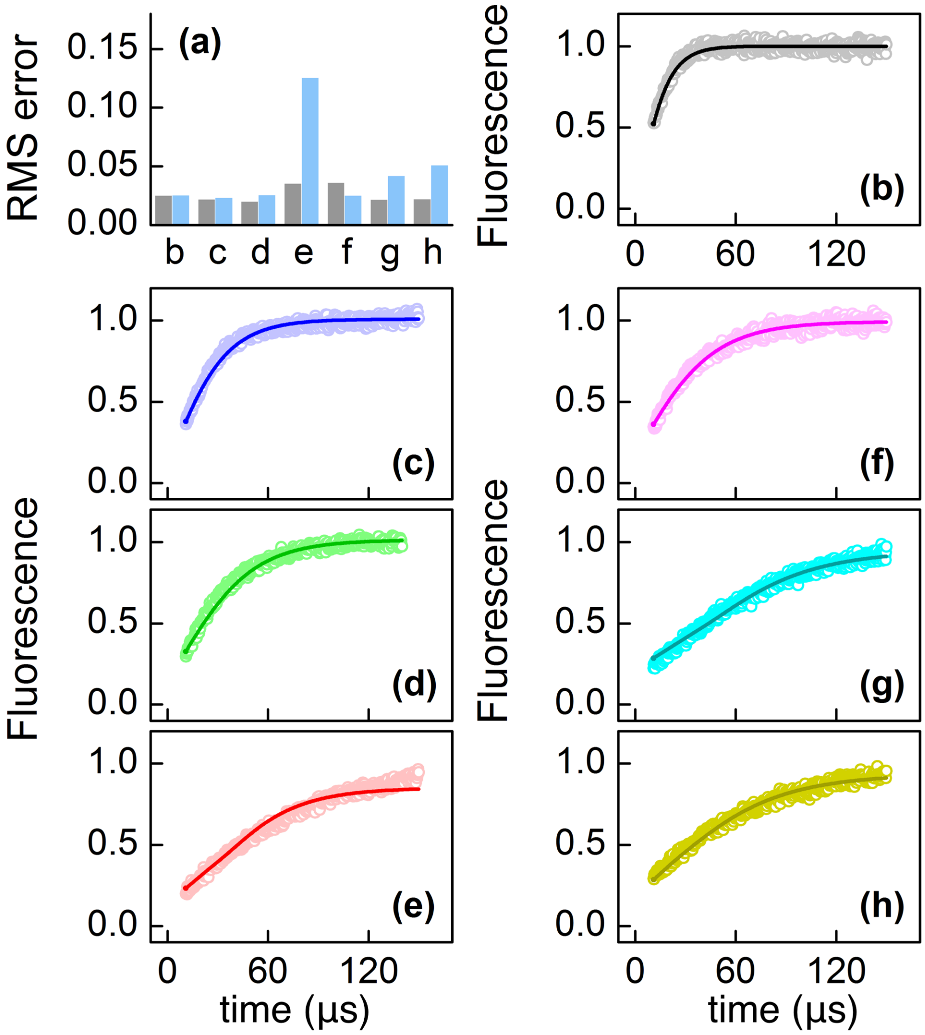

Supplementary Figure 23 | Fitting of fluorescence considering the DMSO concentration gradient. (**a**) Root mean square (RMS) errors of theoretical fitting with respect to experiment in normalized fluorescence intensities for the seven experimental systems in our study (**b-h**). The results of theoretical fitting from the original model with constant DMSO concentration (in grey) and a modified model considering DMSO concentration change along the microfluidic tube (in blue) are compared. (**b-h**) shows the fluorescence measured by the experiments (light dots) and predicted by the theory considering DMSO concentration change (solid curves). The black, blue, green and red curves in (**b-e**) represent the systems in the same solvent (with 0.16 mole fraction of DMSO) but with different initial HPS concentrations of 6 mM, 4 mM, 3 mM and 2 mM, respectively; while the black, magenta, cyan and yellow curves in (**b**, **f-h**) represent the systems with the same HPS initial concentration (6 mM), but with different DMSO mole fractions of 0.16, 0.21, 0.26 and 0.32, respectively. To obtain converged numerical solutions, the first $5\mu s$ of experimental fluorescence data was not included in the fitting. Please refer to supplementary note 8 for more details of the modified model considering DMSO concentration changing along the microfluidic tube.

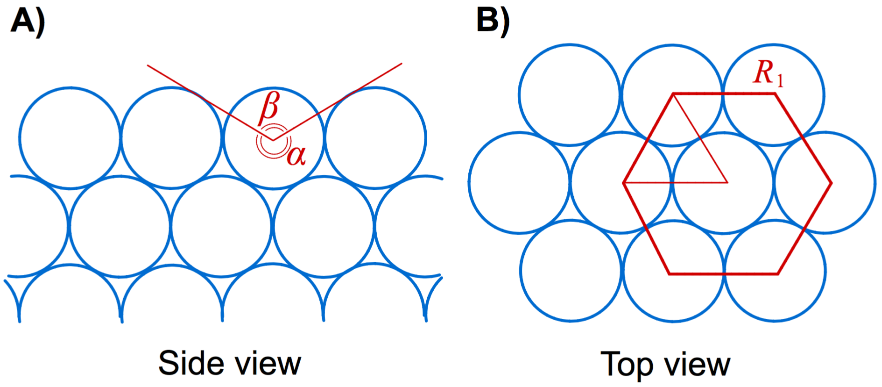

Supplementary Figure 24 | Illustration of the closely packing of large aggregates. As the radius of the aggregate is significantly larger than the radius of a molecule, the surface of the aggregate could be treated as a planar for molecules on the surface of the aggregate. (**a**) The side view shows the solid angle α occupied by the buried part of the molecule within the aggregate. (**b**) The top view illustrates the packing of the molecules on the surface of the aggregate.

Supplementary Table 1. List of surface tensions of various DMSO/water solvent mixtures, viscosities of various DMSO/water solvent mixtures, and diffusion coefficients of HPS molecule in various DMSO/water solvent mixtures. All calculations were performed at a temperature of 293 K.

DMSO mole fraction	γ_{lv} $(\mathrm{cal \cdot mol^{-1} \AA^{-2}})$	Viscosity $(\mathrm{mPa \cdot s})$	HPS molecule diffusivity $(10^4\ \mathrm{\AA^2} \cdot \mu s^{-1})$
0	104.8	1.00	3.46
0.162	84.9	2.45	1.39
0.208	82.5	2.90	1.17
0.26	80.3	3.32	1.03
0.321	78.0	3.61	0.94

Supplementary Table 2. List of solute-solvent surface tension of HPS aggregates (γ_{sl}), critical nucleus size, nucleation barrier, surface energy of HPS aggregates ($KT \cdot \theta$), and free energy h in various DMSO/water solvent mixtures.

DMSO mole fraction	γ_{sl} ($\mathrm{cal \cdot mol^{-1} \cdot Å^{-2}}$)	Critical nuclei size	Nucleation barrier ($\mathrm{kcal \cdot mol^{-1}}$)	KT·θ ($\mathrm{kcal \cdot mol^{-1}}$)	h ($\mathrm{kcal \cdot mol^{-1}}$)
0	31.8 (fitted)	2	16.7	17.7 (fitted)	13.6 (fitted)
0.162	20.9 ± 0.6	3	18.8	11.60	8.52
0.208	19.5 ± 0.6	4	22.7	10.80	7.78
0.26	18.5 ± 0.6	5	25.8	10.25	7.18
0.321	17.0 ± 0.6	6	27.4	9.44	6.75

Supplementary Note 1. The design, fabrication, implementation, and characterization of microfluidic mixer

Microfluidic mixer design, fabrication and implementation. The microfluidic mixer was first designed by Knight *et al.*[2], optimized by Hertzog *et al.*[3, 4], and further improved by Yao *et al*.[5, 6]. We adopted the mixer design by Yao *et al*.[5, 6]. The mixer was fabricated as follows: A photoresist (HPR 504) coated, 525-μm-thick silicon wafer was: 1) photolithographyly patterned with designed mixers; 2) developed by a positive photoresist developer (FHD-5); 3) etched by deep reactive-ion etching (DRIE) of silicon at a depth of 10 ± 0.3 μm; 4) photoresist stripping. Supplementary Figure 9a shows the SEM image of the etched microchannels at the intersection for fluid mixing. The smallest widths of nozzles at the center inlet channel, two side inlet channels, and exit channel in our fabricated mixers are 2.2 ± 0.2 μm, 2.2 ± 0.2 μm, and 2.7 ± 0.2 μm, respectively. Then, a ~2500 Å thermal silicon oxide layer (as a protection layer in the downstream processes) was created on the surface of the wafer by reacting with oxygen after heating the wafer at 1000 °C in a diffusion furnace. The patterns of liquid access holes were transferred on the inlets and outlets of the mixers, followed by tetramethylammonium hydroxide (TMAH) etching through the silicon wafer and buffer oxide etching (BOE) of the silicon oxide layer. Finally, microchannels were sealed by anodic bonding with a 170-μm-thick Pyrex glass (SENSOR Prep Services, Inc.) and individual chips were diced using a wafer cutting machine.

In microfluidic experiments, the mixer was mounted on a chip holder and integrated into the laser scanning confocal microscope (Zeiss, LSM710). Hexaphenylsilole (HPS) was dissolved in pure dimethyl sulfoxide (DMSO) at a concentration of 6, 4, 3, or 2 mM and driven into the center inlet. Simultaneously, a mixture of DMSO and water at a volume ratio of 1/9, 2/8, 3/7, or 4/6 was driven into two side inlets. The flow rates at each inlet were controlled by compressed air above liquid reservoirs and regulated by LabVIEW (National Instruments) operated pressure transducers (Marsh Bellofram Type 2000, Newell, WV). In all microfluidic experiments, the volumetric flow rates of sample solution and solvent mixture were ~2.8 $\mu L \cdot h^{-1}$ and ~280 $\mu L \cdot h^{-1}$, respectively. Such flow conditions generated a laminar flow with a maximum flow velocity of ~1 $m \cdot s^{-1}$ at the center of exit channel. Then, the aggregation of HPS in mixer was monitored under the laser scanning confocal microscope (Zeiss, LSM710). Briefly, the solution of HPS molecules dissolved in DMSO (indicated as red stream in Figure 1b) is continuously pumped into the center microchannel. This central stream is hydrodynamically squeezed by two side water streams to form an extremely narrow stream with tens of nanometers in width. Hence, rapid solvents exchange occurs in a pure diffusion manner, and the immediate environment for HPS aggregation is reached within a few microseconds. In the mixer, the time course of HPS aggregation was determined by dividing the travelling distance of mixture solution along the exit microchannel by its flow velocity. Thus, the progress of HPS aggregation in downstream can be monitored by the integrated confocal system at submicrosecond temporal resolution. Fluorescence images were captured with spatial resolutions of 1 μm and 0.5 μm in vertical (depth) and horizontal (width) directions, respectively. A diode laser at 405 nm was employed as the excitation source. The excitation beam was focused into the center layer of microchannels by an oil immersion objective lens (63×/1.4 NA). Then, fluorescence was collected by the same objective and detected by a photomultiplier tube (PMT) detector at wavelength of

420-600 nm. Figure 2a shows a representative captured image with subtraction of background fluorescence.

Computational fluid dynamics simulations. The fluid flow and mixing dynamics of the center stream sheathed by two adjacent streams in the mixer were simulated using computational fluid dynamics (CFD) software package, COMSOL Multiphysics 4.1. CFD simulation has been shown to be a robust and reliable method to quantify mass transfer in fluidic system. During our CFD simulations, essential parameters, including DMSO's diffusion coefficient, fluid viscosity, fluid density, and their variations in response to the changes of fluid composition have all been taken into account to obtain the DMSO concentration gradient along the microfluidic tube. This numerical simulation approach has been well validated both qualitatively (e.g. by us[7] and Jahn et al.[8]) and quantitatively[6] in various solvent mixtures. Fluid flow inside microchannels is governed by the incompressible steady-state Navier-Stokes equations and the mixing dynamics is governed by the steady-state convective-diffusion equation:

$$\nabla \cdot \mathbf{v} = 0 \tag{1}$$

$$\rho(\mathbf{v} \cdot \nabla)\mathbf{v} = -\nabla P + \mu \nabla^2 \mathbf{v} \tag{2}$$

$$\mathbf{v} \cdot \nabla C = D \nabla^2 C \tag{3}$$

where $\mathbf{v}$ is the flow velocity, ρ is the fluid density, P is the pressure, μ is the fluid dynamic viscosity, C is the molar concentration, and D is the mutual diffusion coefficient. In the simulation, the fluid density, dynamic viscosity, and diffusion coefficient were a function of the DMSO molar concentration and were expressed by a fourth-order polynomial fitting density data, viscosity data, and diffusion coefficient data as reported in literatures[9, 10]. To simulate the flow and diffusion of DMSO and water in the mixer, a symmetric 2D model of the mixer with meshing elements was created as illustrated in Supplementary Figure 9b. The model contains two inlets boundary, one outlet boundary, one symmetric boundary, and the other surface wall boundaries. The initial conditions were set according to the experimental conditions described above.

To quantify the DMSO depletion dynamics in the mixer, we traced 20 streamlines from the sheathed central stream in our 2D model. We followed previous work[4, 7, 11] to define the depletion time t_i of each streamline in our system, of which the timer starts when the molar concentration of DMSO along the streamline has decreased to the 99% of the initial concentration in the central stream. The average time of DMSO depletion $\langle t(x) \rangle$ at x location was calculated by summing and averaging the contribution of the depletion times from all streamlines:

$$t_i(x) = \int_{x_0, c=0.99c_0}^{x} \frac{dx}{u_i(x)} \tag{4}$$

$$\langle t(x) \rangle = \frac{\sum_i t_i(x) w_i(x)}{\sum_i w_i(x)} \tag{5}$$

The standard deviation of the mixing time, which represents the mixing uniformity across the x location, is defined as:

$$\sigma(x) = \sqrt{\frac{\sum_i \left(t_i(x) - \langle t(x) \rangle\right)^2 w_i(x)}{\sum_i w_i(x)}} \qquad t_i > 0 \tag{6}$$

where u_i is the x component of the flow velocity in the ith streamline, w_i is the width of the ith streamline, and C_0 is the initial molar concentration of DMSO. All the simulated data were analyzed using customer-built programs in MATLAB.

Supplementary Figure 9c shows the representative simulation results with a DMSO/water volume ratio of 1/9 in the side stream. The DMSO depletion dynamics along the sheathed central stream at various DMSO/water volume ratio conditions are plotted in Supplementary Figure 9d. The results show that the mole fractions of DMSO deplete rapidly within ~4 ± 1 μs at all conditions, and reach a relative steady-state mole fraction of 0.16, 0.21, 0.26, and 0.32, at conditions with DMSO/water volume fraction of 1/9, 2/8, 3/7, and 4/6 in the side stream, respectively.

Supplementary Note 2. Solubility of HPS and fluorescence intensity of HPS aggregates in bulk solution

Materials preparation. HPS was prepared according to the published procedures[12]. The solutions of HPS with a concentration of 6.0, 4.0, 3.0, 2.0, and 0.1 mM were prepared by dissolving an appropriate amount of the luminogen in DMSO. All experiments were performed at 20 °C unless otherwise specified. An appropriate amount of DMSO was added to the water to achieve different DMSO mole fractions. An appropriate amount of HPS solution was added to the water/DMSO mixture with different final luminogen concentrations. The mixtures were incubated at 20 °C overnight before fluorescence measurement and particle size analysis.

HPS solubility measurements by particle size analysis. We used particle size analysis to estimate HPS solubility. Briefly, an appropriate amount of HPS stock solution was added to the water/DMSO solvent mixture (DMSO mole fraction of 0.16, 0.21, 0.26, or 0.32) with final HPS concentrations ranges from 0.1 to 20 μM. The mixture solutions were incubated overnight before subjecting to particle size analysis (ZetaPALS, Brookhaven Instruments Corporation, U.S.A.). As shown in Supplementary Figure 10, the solubility of HPS molecule was determined to be 0.6 ± 0.1, 2.5 ± 0.2, 6.0 ± 0.5, and 12.5 ± 0.5 μM in the solvent mixture with a DMSO mole fraction of 0.16, 0.21, 0.26, and 0.32, respectively.

Fluorescence spectrofluorometer measurements. Steady-state fluorescence spectra were recorded on a Perkin-Elmer LS 55 spectrofluorometer with a Xenon discharge lamp excitation (at an excitation wavelength of 405 nm). Supplementary Figure 2 shows the emission spectra of HPS aggregates at different HPS concentrations in the solvent mixture with DMSO mole fraction of 0.16. The emission maximum for all conditions is ~ 510 nm. Supplementary Figure 3 shows the fluorescence intensity (at 510 nm) of HPS aggregates in solutions at various HPS concentrations and various DMSO mole fractions. The linear relationships between HPS concentrations and their fluorescence at various solvent mixtures were obtained. This linear relationship suggests that the total volume of aggregated HPS is proportional

to their total fluorescence intensity under the condition of the same solubility of HPS in the same solvent condition.

Fluorescence confocal atomic force microscopy measurements. We also used laser scanning confocal microscope (Leica TCS-SP5) coupled with atomic force microscopy (VeecoBioScope II Catalyst module) to simultaneously measure the volume and fluorescence intensity of individual HPS aggregate. A drop of 20 μL HPS solution at a concentration of 1 μM in pure water was pipetted on an ultrasonic cleaned cover glass. After the solution dried at room condition, AFM measurements were performed in an air-tapping mode to determine the volume of individual aggregate. AFM images were recorded at a scan rate of 0.5 Hz and image size of 40 × 40 μm^2 with 640 × 640 points (Supplementary Figure 4 left inset). Using the laser scanning confocal microscope equipped with a DPSS laser operated at the excitation wavelength of 405 nm, a fluorescence image (with detection wavelength of 420-600 nm) was captured at the corresponding area to determine the fluorescence intensity of individual aggregates (Supplementary Figure 4 right inset). Supplementary Figure 4 shows the experimental data indicated by open circle. Co-plotted red solid line is a linear fitting of experimental data. Again, a linear relationship between the volume of aggregate and its fluorescence intensity was observed.

Small aggregates (size below 20) have negligible contributions to measured fluorescence. Up to this note, we have provided three types of evidence to support the assumption that the total aggregate volume scales linearly with fluorescence intensity: AFM experiment (Supplementary Figure 4), spectrophotometer experiments (Supplementary Figure 3), and quantum mechanics/molecular mechanics (QM/MM) calculations[13] (Supplementary Figure 1). We note that both AFM and bulk spectrophotometer experiments were conducted under the condition of significantly larger HPS aggregate size compared to that in the microfluidic experiments. The QM/MM calculations show that when aggregates reach size 20 or larger, the fluorescence quantum efficiency (FQE) remains nearly invariant, indicating a linear relationship between aggregate volume and fluorescence intensity (Supplementary Figure 1c). For small aggregates with size below 20, it is not clear if this linear relationship would hold. To address this issue, we computed the fraction of small aggregates (size below 20) in total aggregate volume when detectable fluorescence is present, and show that they have little impact: < 3% (Supplementary Figure 13 b1-b7). This is consistent with the fact that in our experiments, we can only begin to detect fluorescence after ~6 microseconds, at which time the averaged aggregate size has already reached ~70, a size that is significantly larger than 20 (under all HPS concentrations and DMSO fractions). Therefore, these small aggregates have negligible contributions to the fitting results.

Supplementary Note 3. Fitting measured aggregation kinetics to the classical nucleation-growth model.

We fitted the experimental measured aggregation kinetics to a theoretical model (Figure 2b and Figure 2c), which was constructed from the classical nucleation-growth theory described above. In this model, new nuclei are being formed at each aggregation time point, and simultaneously existing aggregates are growing. Once new nuclei are formed, they continue to grow as long as the solution is supersaturated.

Accordingly, the total amount of aggregated HPS per unit volume at time t is the integration of the product of nuclei generated at a previous time τ_J and its size growing during the remaining time τ_G:

$$V(t) = \int_0^t J(s)\left(n^*(s) + \int_s^t \frac{dg(\tau', s)}{d\tau'} d\tau\right) ds \tag{7}$$

where $J(s)$ is the number of nucleus generated in unit volume per time, $n^*(s)$ is the critical nucleus size, and $dg(\tau', s)/d\tau'$ is the growth rate of the nucleus that formed at time s. Meanwhile, the total amount of aggregated HPS can be independently calculated from supersaturation ratio $S(t)$:

$$\frac{V(t)}{V_{\mathrm{T}}} = \frac{C_1(0) - C_1(t)}{C_1(0)} = \frac{S(0) - S(t)}{S(0)} \tag{8}$$

where V_{T} is the total amount of HPS molecules in the unit volume, $C_1(0)$ is the initial concentration of HPS in the solution, $C_1(t)$ is the monomer concentration at time t, and $S(0)$ is the initial supersaturation ratio. We notice that supplementary equation (7) and supplementary equation (8) are self-consistent, so that with a given initial concentration $C_1(0)$, the solution of $V(t)$ can be obtained numerically.

On the other hand, we showed that the total amount of aggregated HPS is linearly correlated with the total fluorescence intensity (Supplementary Figure 1 to 4). This correlation allows us to establish a connection between the measured fluorescence intensity in experiments and the total amount of aggregated HPS $V(t)$ in the theoretical model. As we proved that the total volume of aggregated HPS is proportional to the total fluorescence intensity, the measured dynamics of normalized fluorescence $I(t)/I_{\mathrm{T}}$ directly reveal the HPS aggregation kinetics $V(t)/V_{\mathrm{T}}$. Therefore, we used the normalized total aggregates volume $V(t)/V_{\mathrm{T}}$ to directly fit the measured normalized fluorescence intensity (Figure 2b and Figure 2c). In particular, the solute-solvent surface tension γ_{sl}, which is the surface tension of HPS aggregates in the solvent mixture, is the only fitting parameter. All fitted curves display strong agreement between experiment and theory, with the Pearson correlation coefficients being larger than 0.95 (Supplementary Figure 11). We note that it takes a few microseconds for the fluid mixing to complete in a microfluidic mixer, and during this premixing process[4, 6], nucleus may already started to form. To take into account of this, we have shifted the aggregation time of experiments during fitting. With the fitted surface tension γ_{sl}, we computed the critical aggregate size n^* and the free energy barrier $W(n^*)$ at the initial nucleation stage $S(0)$ using equation (3) and equation (2) respectively, and the results are summarized in Supplementary Table 2.

Supplementary Note 4. The non-classical nucleation and growth theory

As our quantitative analysis heavily relies on the CNT, we have examined an alternative theory: a non-classical nucleation theory (non-CNT) involving stable prenucleation clusters[14, 15]. We constructed the non-CNT theory based on the Dillmann-Meler model[14] that involves two free energy barriers: the critical nucleation barrier and a second barrier ε,

$$\Delta G(n) = \theta n^{2/3} - nh + \varepsilon \tag{9}$$

Where the first two terms are identical with CNT, while ε corresponds to the effective barrier for the transition from nucleated amorphous clusters to the crystalline phase per molecule[16]. For a simple

approximation, we treat ε as a constant. This second free energy barrier ε will introduce corrections on both nucleation rate (J) and growth rate (f) (Following a similar derivation in the kinetic nucleation theory[17]):

$$J_{\mathrm{non-CNT}} = e^{-\varepsilon} J_{\mathrm{CNT}} \quad (10)$$

$$f_{\mathrm{non-CNT}} = e^{-\varepsilon} f_{\mathrm{CNT}} \quad (11)$$

When ε equals 0, the non-CNT theory is reduced to the CNT theory. When $\varepsilon \gg kT$, the second free energy barrier becomes the rate-limiting step (e.g. for protein systems ε can be as large as 20 kT so that the nucleation rate is $\times 10^9$ lower than the predictions of CNT[18].

We show that ε has to be within 1.2 kT in order to obtain reasonable fitting to experimental data. After applying nucleation and growth rates defined in Eq. 24 and 25 to our model, we examined the quality of model fitting to experimental fluorescence over a wide range of ε (0, 0.1, 0.5, 1.2, 2.3, 3.0, 3.9, and 4.6 kT). As shown in Supplementary Figure 14, when $\varepsilon > 1.2kT$, the non-CNT model provides very slow aggregation rates and the final aggregate size is still within 15. As suggested by our previous work Ref[1], aggregates with this small size will have majority of the HPS molecules exposed to solvent with low quantum yields, and thus may not be able to emit detectable fluorescence. Consistently, we notice that the relative deviations of theoretical fitting from experiment (on the average slope of the total fluorescence intensity) become more significant with the increase of ε among all HPS initial concentrations (see Supplementary Figure 15). Therefore, ε has to be within $1.2kT$ in order to obtain reasonable agreement between the non-CNT theory and experiment.

When $\varepsilon \leq 1.2$ kT, the second free energy barrier is at the order of thermal fluctuations or even smaller, which should not have substantial impact on the nucleation process. Indeed, we show that the predictions of non-CNT theory match well with those from the CNT theory in both ΔΔG and cooperativity for different systems (see Supplementary Figure 16). Therefore, we conclude that the CNT theory can sufficiently well describe the HPS aggregation process in this study. In the literature, the non-CNT theory is found to be particularly useful when describing systems such as crystallization of ionic compounds and proteins[15]. We anticipate that hydrophobic interactions that drive HPS aggregation is non-specific in nature, and this is in contract to the formation of specific ion pairs during the crystallization of ionic compounds. Therefore, the HPS aggregation process may not favor the formation of prenucleation clusters as much as ionic compounds. In addition, as HPS is more rigid compared to flexible polymers like proteins, a second free energy barrier due to the re-arrangement of protein upon initial collapse is not likely to be present in our HPS system.

Supplementary Note 5. An alternative way of estimating pair potential of mean force by umbrella sampling molecular dynamics simulations

In this study, accurately estimating the pair potential of mean force (δF) is important for the measure of cooperativity. In our CNT model, we estimated δF as averaged pair interactions in smallest stable aggregates (size 3-7) using Supplementary Eq. 14. To further validate the value of δF, we have adopted an alternative computational approach to directly compute δF. In particular, we performed molecular dynamics (MD) simulations with umbrella sampling to compute the potential of mean force (PMF) for

bringing together a pair of HPS molecules in water solution (~1100-ns simulations in total, see Supplementary Figure 17). From MD simulations, we obtained $\delta F = -1.30 \pm 0.15\ \mathrm{kcal \cdot mol^{-1}}$ per molecular contact. The mean value of δF is slightly weaker than our previous estimation based on Supplementary Eq. 14 ($-1.33\ \mathrm{kcal \cdot mol^{-1}}$), even though these two values are well within the uncertainty of computation. Subsequently, we show that the cooperatively is also slightly increased by ~2% if we adopt the δF value estimated from MD simulations (Supplementary Figure 18). These results suggest that δF computed from the CNT theory and MD simulations are in reasonable agreement in predicting cooperativity associated with the HPS aggregation.

We have followed the same protocol as described in our previous work[1] to set up MD simulations except that these simulations were performed at 293K to match our experimental temperature. In umbrella sampling, we have applied harmonic potentials (with the force constant of $4.78\times10^{4}\ \mathrm{kcal \cdot mol^{-1} Å^{-2}}$) to restrain the pair of HPS molecules to be at specific distance windows (i.e. 5, 6, 7, … 20 Å in Si-Si distance). For windows where PMF converges fast ($r < 5$ Å or $r > 15$ Å), we performed a 20-ns simulation for each distance, while for windows where PMF converges slow (5 Å $\leq r \leq$ 15 Å), we performed a 40-ns simulation per distance. We then applied the Weighted Histogram Analysis Method (WHAM)[19] to remove bias due to these restrain potentials, and obtained the PMF curves. When performing the WHAM analysis, we have removed the first 1-ns simulation for equilibration. All our simulations were performed using the Gromacs software[20].

In addition, we noticed that all-atom representations of HPS in our MD simulations is not perfectly spherical; as such, this will subsequently affect its packing in HPS aggregates. In particular, each contact of two HPS molecules in MD simulations contains different number of contacting atom pairs. Specifically, averaged number of atomic contacts per molecular contact in simulations of HPS dimer, aggregates of size 10, 40, 50 and 60 are 22.4, 9.1, 7.7, 7.5 and 7.2, respectively. To obtain these numbers, 10,000 MD snapshots of aggregates with each size taken from Ref[1] were included in the calculations. For each MD snapshot, the *g_contacts* algorithm[21] was adopted to compute number of intermolecular atomic contacts (carbon atom were included in calculations and the cutoff distance was set to be 4 Å). From the dimer PMF curves, we can then estimate the average strength of individual atomic contact (by dividing 22.4), and further obtain effective PMF per molecular contact in aggregates (e.g. aggregates of size 60 contains 7.2 atomic contacts per molecular contact). As the averaged atomic contact number remain nearly invariant for aggregates of size larger than 40, we applied an atomic contacts/molecular contact of 7.2 to the PMF curves reported in Supplementary Figure 17. To ensure the convergence of conformational sampling, we have performed two independent sets of simulations: one was bringing two HPS molecules together from 20 to 5 Å, while the other one was separating two molecules apart from 5 to 20 Å. As shown in Supplementary Figure 17, these two independent sets of simulations generated PMF curves that were in reasonable agreement. To extract δF from the PMF curves, we computed Boltzmann weighted average of PMF values in the first free energy minimum (6.4Å $\leq d_{Si-Si} \leq$ 8.4Å).

Based on the PMF curves, we obtained $\delta F = -1.30 \pm 0.15\ \mathrm{kcal \cdot mol^{-1}}$ per molecular contact. This value suggests a slightly weaker pair interaction compared with our previous estimation based on the smallest stable aggregates using the CNT theory ($\delta F = -1.33\ \mathrm{kcal \cdot mol^{-1}}$). Nevertheless, the two

estimations are within the uncertainty of computation. As shown in Supplementary Figure 18, the resulting cooperativity also increases slightly by ~2%, even though the CNT theory prediction is well within the standard deviation of MD simulation results. We note that the free energy calculations based on MD simulations and force fields often introduce errors of around 0.5 to 1 $\text{kcal} \cdot \text{mol}^{-1}$ even for the hydration of small chemical compounds[22]. In summary, we show that an alternative way of obtaining δF from MD simulations provide consistent results with our CNT theory.

Supplementary Note 6. The effect of exposed HPS molecules with weak fluorescence emission on our model fitting

QM/MM calculations show that the ability for exposed molecules in HPS aggregates to emit fluorescence is significantly weakened (see FQE plot in Supplementary Figure 19(a)). In this note, we examine if weak emissions from these exposed HPS molecules will have significant effects on the results of our model. To examine the impact of exposed HPS molecules, we revised our model so that only buried HPS molecules can emit fluorescence, which results in a smaller effective aggregate size:

$$n_{\text{buried}} = \left(n^{1/3} - 1\right)^3 \tag{12}$$

By fitting the total volume of buried HPS molecules to experimental fluorescence, we show that this modified model only considering buried HPS molecules can also fit well to the experimental fluorescence (Supplementary Figure 19(b)), and the fitted $\Delta\Delta G$ and cooperativity also match well with our original model considering all HPS molecules in aggregates (Supplementary Figure 20). These results suggest that there exists a constant scaling factor between fluorescence curves computed based on all molecules and buried ones. To further examine this scaling factor, one may need to know total number of absorbed photons in experiment. Nevertheless, both models generate consistent results in the strength of hydrophobic interactions and magnitude of cooperativity.

Supplementary Note 7. Connecting with the LCW theory

Lum, Chandler, and Weeks (LCW) predicted in their theory[23] that the differences between small-scale versus large-scale in hydrophobic interactions arise from a crossover from volume-based hydration thermodynamics for small solutes (< 1 nm in radius) versus area-based thermodynamics for large solutes (>1 nm in radius). They suggested that this crossover is related to the persistence of the hydrogen-bonding network. For example, a drying transition may occur around large non-polar solutes due to an energetic effect, given that the persistence of the hydrogen bond network becomes geometrically impossible[23]. A more direct explanation for the crossover of the hydrophobic effect is that waters around large non-polar solutes undergo significant non-Gaussian density fluctuations, and thus render them in proximity to a liquid-vapor phase transition.

If this crossover exists, we should observe a kink when the solvation free energy per surface area ($\Delta G/\text{Å}^2$) is plotted against the radius of the solute as proposed in Figure 2 of Ref [24]. Furthermore, the magnitude of the cooperativity should be correlated with the difference in $\Delta G/\text{Å}^2$. From our model, the solvation free energy can be obtained as $\Delta G_A = \theta n^{2/3}$ for aggregate of size n, and $\Delta G_A = \phi$ for an

individual molecule. The solvent accessible area A for an individual HPS is given in Supplementary Note 3, and the solvent accessible area A for aggregates can be computed by

$$A(n \geq 2) = 4\pi(R_n + R_{\mathrm{W}})^2 = 4\pi\left(\frac{n^{1/3}R_1}{0.7^{1/3}} + R_{\mathrm{W}}\right)^2 \qquad (13)$$
$$A(n = 1) = 750\ \text{Å}^2$$

Where the solvent accessible area of monomer in obtained from Supplementary Note 3. $R_1 = 5.9$ Å is the radius of the HPS molecule, $R_{\mathrm{W}} = 1.4$ Å is the radius of water. As shown in Supplementary Figure 21, we plotted the $\Delta G/\text{Å}^2$ for the HPS aggregation in four different DMSO/water mixture solvents. Indeed, there exists a clear kink before and after the crossover length scale at around 1 nm as suggested by the LCW theory. More interestingly, the difference in $\Delta G/\text{Å}^2$ before and after the kink clearly increases with the increase of water fraction in the solvent mixture, indicating stronger cooperative part of the formation energy in solvent mixtures containing more water.

Supplementary Note 8. A modified CNT model considering the DMSO concentration change along the microfluidic tube

In our original CNT model, we treat the DMSO concentration as a constant. However, as shown in Supplementary Figure 9d, the DMSO concentration profiles display noticeable drift along the microfluidic tube. To examine the impact of this DMSO concentration variation, we have modified our theory to incorporate the DMSO concentration variation along the microfluidic jet. To achieve this, we first fitted each DMSO concentration profile ($C_{\mathrm{DMSO}}(t)$) with a double-exponential function (Supplementary Figure 9d). We then applied these functions of DMSO concentrations to our nucleation growth model. In particular, we first obtained the liquid-vapor surface tensions (γ_{lv} or $\gamma_{solvent}$) of solvent mixtures containing different mole fractions of DMSO from previously published results (see Figure 1 of Ref [10]). We can then obtain the DMSO concentration dependence of the phase transfer free energy ($-h$) by interpolating a linear function fitted to Supplementary Figure 5a. We can then obtain super-saturation ratio (S), and all other quantities depending on these parameters become DMSO concentration dependent. Our classical nucleation and growth theory can then be modified as:

$$W(n) = \theta n^{2/3} - n \cdot (kT \cdot \ln S(t) + \Delta h) \qquad (14)$$

$$n^* = \left(\frac{2\theta}{3kT \ln S(t) + 3\Delta h}\right)^3 \qquad (15)$$

$$J(t) = \left[(36\pi)^{1/6} \frac{DC_e}{\sqrt[3]{v^2/p^2}\sqrt{\theta/kT}}\right] S(t) e^{-\Delta h/kT} e^{\frac{-4\theta^3/kT}{27\,(kT \ln S(t) + \Delta h)^2}} \qquad (16)$$

$$\frac{dg(t)}{dt} = 4\pi D C_e \left[\frac{3nv}{4\pi p}\right]^{1/3} \cdot \left[S(t) - e^{-\Delta h/kT}\right] \qquad (17)$$

Where $\Delta h = h(t) - h$ is the correction on previously applied h. As shown in Supplementary Figure 23, the modified CNT theory considering DMSO concentration gradient produced consistent results with our original model in both hydrophobic free energies ($\Delta\Delta G$) and cooperativity (Supplementary Figure 23). These results suggest that taking into account the DMSO concentration gradient does not have significant impact on the fitting results. To further explain this, we show that $-h$ only has minor shift (~2%) when

the DMSO concentration is allowed to vary, and the $-h$ value from our previous model lies well within the standard deviation of that from this modified model (Supplementary Figure 23a). As a result, $\Delta\Delta G$ predicted from our original model is also well within the standard deviation of the modified model when considering DMSO concentration gradient (Supplementary Figure 23b). For the modified model, we also noticed that the quality of fitting is noticeably reduced for certain systems (e.g. larger root mean square errors for system e, g and f as shown in Supplementary Figure 24) in comparison to the original model, which may be due to the increase of numerical complexity upon the introduction of a double-exponential function for DMSO concentrations. Moreover, converged numerical solutions could only be obtained when we leave out first $5\mu s$ of experimental fluorescence data. We anticipate that during this initial time window, the system is still not thoroughly mixed and is still undergoing drastic changes of the DMSO concentration, and thus may not be well described by our model.

Supplementary Note 9. Proof of equation (12)

To compute the number of contact pairs in an aggregate containing n molecules, we distinguished the molecules enclosed inside the aggregate from those on the surface of the aggregate. For a molecule that is enclosed in a sufficiently large aggregate, the number of contact pairs for this molecule is $P_{\mathrm{mon}} = 6$. To compute the number of contact pairs of a molecule on the surface of the aggregate, we assumed that the number of contact pairs between a molecule and other molecules is proportional to the solid angle α occupied by the buried part of the molecule inside the aggregate. For a molecule enclosed inside the aggregate (totally buried), the solid angle α equals 4π. For a molecule on the surface of the infinite sized aggregate as shown in Supplementary Figure 24a (side view of the aggregate), the solid angle α equals 3π. Consequently, the number of contact pairs lost for this molecule at the surface of the aggregate is:

$$P_{\mathrm{loss}} = \left(\frac{4\pi - 3\pi}{4\pi}\right) P_{\mathrm{mon}} = \frac{P_{\mathrm{mon}}}{4} \tag{18}$$

Next, we estimated the number of molecules on the surface of the aggregate. As shown in Supplementary Figure 24b (top view of the aggregate), the surface area occupied by a molecule on the surface of the aggregate is 1/3 of the hexagon highlighted by the red line:

$$A_1' = 2\sqrt{3}R_1^2 \tag{19}$$

where R_1 is the radius of monomer. Therefore, the number of molecules on the surface of the aggregate is:

$$n_{\mathrm{A}} = \frac{A_n}{A_1'} = \frac{4\pi R_1^2 n^{2/3}}{2\sqrt{3}R_1^2 p^{2/3}} \tag{20}$$

where A_n is the surface area of the aggregate, and $p = 0.7$ is the packing density of the aggregate. Combining supplementary equations (18)-(20), we obtained that the number of contact pairs in the aggregate containing n molecules is equation (12):

$$P_n = \left(n - \frac{P_{\mathrm{loss}}}{P_{\mathrm{mon}}} n_{\mathrm{A}}\right) P_{\mathrm{mon}} \approx 6\left(n - 1.2n^{2/3}\right) \tag{21}$$

The above equation is derived for a large aggregate. Next, we showed that equation (12) or supplementary equation (21) is also valid for the small aggregates: for $n = 3, 4, 13$, the number of contact pairs given by equation (12) or supplementary equation (21) is $P_3 = 3.0$, $P_4 = 5.8$ and $P_{13} = 38.2$, respectively. These

values are very close to the number of contact pairs given by the ideal packing: $P_3^{\mathrm{real}} = 3$, $P_4^{\mathrm{real}} = 6$ and $P_{13}^{\mathrm{real}} = 36$. Hence, we prove that equation (12) or supplementary equation (21) is a good approximation for the number of contact pairs in both small and large aggregates.

Supplementary References

1. Zheng, X. *et al.* Unraveling the aggregation effect on amorphous phase AIE luminogens: a computational study. *Nanoscale* **8**, 15173-15180 (2016).
2. Knight, J.B., Vishwanath, A., Brody, J.P. & Austin, R.H. Hydrodynamic focusing on a silicon chip: mixing nanoliters in microseconds. *Phys. Rev. Lett.* **80**, 3863 (1998).
3. Hertzog, D.E. *et al.* Femtomole mixer for microsecond kinetic studies of protein folding. *Anal. Chem.* **76**, 7169-7178 (2004).
4. Hertzog, D.E., Ivorra, B., Mohammadi, B., Bakajin, O. & Santiago, J.G. Optimization of a microfluidic mixer for studying protein folding kinetics. *Anal. Chem.* **78**, 4299-4306 (2006).
5. Jiang, L. *et al.* Microsecond protein folding events revealed by time-resolved fluorescence resonance energy transfer in a microfluidic mixer. *Anal. Chem.* (2015).
6. Yao, S. & Bakajin, O. Improvements in mixing time and mixing uniformity in devices designed for studies of protein folding kinetics. *Anal. Chem.* **79**, 5753-5759 (2007).
7. Jiang, L.G., Wang, W.P., Chau, Y. & Yao, S.H. Controllable formation of aromatic nanoparticles in a three-dimensional hydrodynamic flow focusing microfluidic device. *Rsc Adv* **3**, 17762-17769 (2013).
8. Jahn, A. *et al.* Microfluidic Mixing and the Formation of Nanoscale Lipid Vesicles. *Acs Nano* **4**, 2077-2087 (2010).
9. Borin, I.A. & Skaf, M.S. Molecular association between water and dimethyl sulfoxide in solution: a molecular dynamics simulation study. *J. Chem. Phys.* **110**, 6412-6420 (1999).
10. Catalan, J., Diaz, C. & Garcia-Blanco, F. Characterization of binary solvent mixtures of DMSO with water and other cosolvents. *J. Org. Chem.* **66**, 5846-5852 (2001).
11. Park, H.Y. *et al.* Achieving uniform mixing in a microfluidic device: hydrodynamic focusing prior to mixing. *Anal. Chem.* **78**, 4465-4473 (2006).
12. Chen, J. *et al.* Synthesis, light emission, nanoaggregation, and restricted intramolecular rotation of 1, 1-substituted 2, 3, 4, 5-tetraphenylsiloles. *Chem. Mater.* **15**, 1535-1546 (2003).
13. Acharya, H., Vembanur, S., Jamadagni, S.N. & Garde, S. Mapping hydrophobicity at the nanoscale: Applications to heterogeneous surfaces and proteins. *Faraday Discuss.* **146**, 353-365 (2010).
14. Prestipino, S., Laio, A. & Tosatti, E. Systematic improvement of classical nucleation theory. *Phys. Rev. Lett.* **108**, 225701 (2012).
15. Gebauer, D. & Colfen, H. Prenucleation clusters and non-classical nucleation. *Nano Today* **6**, 564-584 (2011).
16. Meldrum, F.C. & Sear, R.P. Materials science. Now you see them. *Science* **322**, 1802-1803 (2008).
17. Kashchiev, D., Vekilov, P.G. & Kolomeisky, A.B. Kinetics of two-step nucleation of crystals. *J. Chem. Phys.* **122** (2005).
18. Vekilov, P.G. The two-step mechanism of nucleation of crystals in solution. *Nanoscale* **2**, 2346-2357 (2010).
19. Mei, J., Leung, N.L.C., Kwok, R.T.K., Lam, J.W.Y. & Tang, B.Z. Aggregation-Induced Emission: Together We Shine, United We Soar! *Chem. Rev.* **115**, 11718-11940 (2015).
20. Hess, B., Kutzner, C., van der Spoel, D. & Lindahl, E. GROMACS 4: Algorithms for highly efficient, load-balanced, and scalable molecular simulation. *J. Chem. Theory Comput.* **4**, 435-447 (2008).
21. Blau, C. & Grubmuller, H. g_contacts: Fast contact search in bio-molecular ensemble data. *Comput. Phys. Commun.* **184**, 2856-2859 (2013).
22. Mobley, D.L. & Guthrie, J.P. FreeSolv: a database of experimental and calculated hydration free energies, with input files. *J. Comput. Aided Mol. Des.* **28**, 711-720 (2014).
23. Lum, K., Chandler, D. & Weeks, J.D. Hydrophobicity at small and large length scales. *J. Phys. Chem. B* **103**, 4570-4577 (1999).
24. Chandler, D. Interfaces and the driving force of hydrophobic assembly. *Nature* **437**, 640-647 (2005).
